# The clinical value of metagenomic next generation sequencing in the diagnosis of non-neutropenic invasive pulmonary aspergillosis

**DOI:** 10.3389/fcimb.2026.1731736

**Published:** 2026-04-14

**Authors:** Huanhuan Zhong, Chao Sun, Yajie Lu, Xiaomin Cai, Min Cao, Li Wang, Chunlai Feng, Mengyue Song, Wenkui Sun, Minhua Shi, Yujian Tao, Jun Zhou, Cheng Chen, Xin Lu, Yuanqin Li, Yueyan Ni, Yuchen Cai, Jinjin Zhong, Yuanyuan Li, Weiwei Wu, Yi Shi, Min Wang, Xin Su

**Affiliations:** 1 Department of Respiratory and Critical Care Medicine, Nanjing Drum Tower Hospital, the Affiliated Hospital of Nanjing University Medical School, Nanjing, China; 2 Department of Respiratory and Critical Care Medicine, Nanjing Jinling Hospital, Medical School of Nanjing University, Nanjing, China; 3 Department of Respiratory and Critical Care Medicine, The Second Affiliated Hospital of Soochow University, Suzhou, China; 4 Department of Respiratory and Critical Care Medicine, Jiangsu Second Chinese Medicine Hospital, Nanjing, China; 5 Department of Respiratory and Critical Care Medicine, Nanjing First Hospital, Nanjing, China; 6 Department of Respiratory and Critical Care Medicine, Changzhou First People’s Hospital, Changzhou, China; 7 Department of Respiratory and Critical Care Medicine, Nanjing Jinling Hospital, Nanjing Medical University, Nanjing, China; 8 Department of Respiratory and Critical Care Medicine, Jiangsu Province Hospital, Nanjing, China; 9 Department of Respiratory and Critical Care Medicine, Affiliated Hospital of Yangzhou University, Yangzhou, China; 10 Department of Respiratory and Critical Care Medicine, The First Affiliated Hospital of Soochow University, Suzhou, China; 11 Department of Respiratory and Critical Care Medicine, Nanjing Jiangning Hospital, Nanjing, China; 12 Department of Respiratory and Critical Care Medicine, The Affiliated Hospital of Xuzhou Medical University, Xuzhou, China; 13 Difei Medical Technology (Nanjing) Co., Ltd., Nanjing, China; 14 Department of Respiratory and Critical Care Medicine, The First Affiliated Hospital, and College of Clinical Medicine of Henan University of Science and Technology, Luoyang, China

**Keywords:** diagnostic value, invasive pulmonary aspergillosis, metagenomic next generation sequencing, microbiological test, non-neutropenic

## Abstract

**Background:**

This study aims to explore the performance of metagenomic next generation sequencing (mNGS) in the diagnosis of non-neutropenic invasive pulmonary aspergillosis (IPA) and its clinical application value.

**Methods:**

This multi-center study enrolled 293 suspected IPA patients who conducted mNGS from October 2020 to February 2024. These cases were classified into IPA group and non-IPA group according to IPA diagnostic criteria. We analyzed the diagnostic value of mNGS by comparing with sputum culture, BALF culture, serum and BALF GM test.

**Results:**

A total of 118 IPA patients (4 proven/113 probable/1 possible diagnosis) were included in our study. The most common *Aspergillus* species was *A. fumigatus* (63.4%), followed by *A. flavus* (23.2%), *A. oryzae* (7.1%), *A. niger* (3.6%) and *A. terreus* (2.7%). The sensitivity of bronchoalveolar lavage fluid (BALF) mNGS was significantly higher than BALF culture (81.9% vs. 27.0%, p<0.001) and BALF galactomannan (GM) (81.9% vs. 55.8% (GM≥1.0 cutoff value), p<0.001). The specificity of BALF mNGS was 92.2%, which was similar with BALF culture (98.5%) and BALF GM (94.7%). The combination of BALF mNGS and GM could increase the sensitivity to 88.7%, and had great negative predictive value (NPV, 92.3%). The sensitivity of blood mNGS was significantly higher than serum GM (58.8% vs. 16.7%, p<0.001). And the sensitivity of sputum mNGS was 66.7%, which was significantly higher than sputum culture (30.0%, p=0.025).

**Conclusion:**

mNGS demonstrated significant diagnostic value for IPA, exhibiting significantly higher sensitivity compared to current conventional microbiological tests while maintaining equivalent specificity. The combination of BALF mNGS with GM performed great sensitivity and negative predictive value. BALF specimens seemed to be superior to blood and sputum samples. However, for patients unable to undergo bronchoscopy, sputum and blood mNGS were still superior to other methods.

## 1 Introduction

Invasive pulmonary aspergillosis (IPA) is a complicated mycosis with high mortality and cost, mainly caused by *Aspergillus fumigatus*, whose conidia are widely present in indoor and outdoor environments (Latgé and Chamilos, 2019). Usually, this fungus can invade immunocompromised host or those with lung diseases, leading to IPA (Latgé and Chamilos, 2019). Previously, IPA was known to occur in neutropenic patients. However, it has been increasingly found that nonneutropenic patients, such as those with chronic obstructive pulmonary disease (COPD), bronchiectasis, previous tuberculosis, diabetes, severe influenza or COVID-19, can also be susceptible to IPA (Latgé and Chamilos, 2019; Bassetti et al., 2021; Donnelly et al., 2020). There are no typical clinical or imaging features in these nonneutropenic patients with IPA, leading to difficulties in early diagnosis (Hosseinikargar et al., 2024; Zarrinfar et al., 2012). Currently, culture and galactomannan test are common conventional test methods according to the guidelines of the European Organization for Research and Treatment of Cancer/Invasive Fungal Infections Cooperative Group and the National Institute of Allergy and Infectious Diseases Mycoses Study Group (EORTC/MSGERC), but these microbiological methods have not achieved satisfactory results in sensitivity and timely diagnosis (Donnelly et al., 2020). Hence, developing new diagnostic assays is urgently needed.

Metagenomic next generation sequencing (mNGS) is a promising technique to analyze the microbial and host genetic content in clinical specimens (Han et al., 2019). Recently, mNGS test with specimens of bronchoalveolar lavage fluid (BALF), blood or sputum has been increasingly used in complicated respiratory infection diseases including IPA. However, due to many reasons including the confusion of vast host genes and microorganism colonization, its diagnostic value has not been fully evaluated. Some studies found that there were outstanding sensitivity and specificity of mNGS in the diagnosis of IPA, but almost limited to small cases or immunosuppressed populations (Ma et al., 2022; Zhan et al., 2023; Ao et al., 2023; Hoenigl et al., 2023). Here, we performed a multi-center prospective study involving patients with suspected IPA to investigate the performance of mNGS in the diagnosis of non-neutropenic IPA and its clinical application value.

## 2 Materials and methods

### 2.1 Study design

This multi-center prospective study, as part of the ‘PTX3 detection of peripheral blood and BALF for the diagnosis of IPA’ study (ChiCTR2200063453), contained hospitalized patients referred to 12 hospitals in Jiangsu province, China from October 2020 to February 2024, who were all suspected IPA and conducted mNGS. All participating units collected data on demographics, clinical and microbiological information, result of mNGS, diagnostic and treatment details by case report form. The 2019 EORTC/MSGERC update criteria was used to identify enrolled patients as IPA or not. The main objective of this study was to explore the diagnostic and clinical application value of mNGS for nonneutropenic patients with IPA by comparing with other microbiological methods.

### 2.2 Inclusion and exclusion criteria

Inclusion criteria: Patients were required to meet all of the following: (1) age>18 years; (2) hospitalized with suspected IPA; (3) underwent mNGS; and (4) provided informed consent. Exclusion criteria: Patients were excluded if any of the following were present: (1) neutropenia; (2) treatment with anti-*Aspergillus* before enrollment; (3) undetermined diagnosis of IPA; (4) allergic bronchopulmonary aspergillosis (ABPA); (5) chronic pulmonary aspergillosis (CPA); (6) refusal to participate in the study.

The definition of ‘suspected IPA’ was identified as: (1) patients had pulmonary or systemic risk factors for IPA including but not limited to chronic obstructive pulmonary disease (COPD), diabetes, long-term use of glucocorticoids or immunosuppressants, solid organ transplantation, malignancy, severe influenza, acute respiratory distress syndrome, malnutrition, near-drowning and so on; (2) patients had the following respiratory symptom, such as fever, cough, expectoration, hemoptysis, chest pain, or dyspnea, and showed no response to empiric antimicrobial therapy; (3) chest imaging showed halo sign, cavity, pulmonary nodules, consolidation, or infiltrates.

### 2.3 Diagnostic criteria for IPA

The diagnostic criteria of IPA were mainly according to the guidelines of the ‘Revision and Update of the Consensus Definitions of Invasive Fungal Disease From the European Organization for Research and Treatment of Cancer and the Mycoses Study Group Education and Research Consortium’ (EORTC/MSGERC 2020) or ‘EORTC/MSGERC Definitions of Invasive Fungal Diseases: Summary of Activities of the Intensive Care Unit Working Group’ (EORTC/MSGERC ICU) (Bassetti et al., 2021; Donnelly et al., 2020). The details were as follows:

1. host factors for IPA, such as hematological malignance, hematopoietic stem cell transplantation (HSCT), solid organ transplant, long term use of immunosuppressive or corticosteroids (≥0.3mg/kg/day prednisone over 3weeks), and some recent new factors including severe influenza or COVID-19, diabetes, COPD, chronic hepatic or renal insufficiency, drowning and so on;
2. clinical and radiological features consistent with IPA, for example, fever, cough or hemoptysis symptoms or dense, well-circumscribed lesions with or without a halo sign, air crescent sign, or cavity in imaging;
3. microbiological evidence of *Aspergillus*, including positive *Aspergillus* culture from sputum or BALF, positive GM detection results of serum or BALF (serum GM≥1.0/BALF GM≥1.0/serum GM≥0.7 and BALF GM≥0.8) or positive *Aspergillus* PCR results;
4. histological evidence of invasive *Aspergillus* hyphae in lung needle aspiration or biopsy specimens or positive *Aspergillus* culture results from sterile site.

The proven diagnosis need meet the fourth criterion only, the probable diagnosis need meet the first 3 criteria, and the possible diagnosis need meet the first 2 criteria and IPA was highly suspected meanwhile. In addition, patients were reassessed by at least two professors of respiratory medicine.

### 2.4 Study population

**Figure 1:**
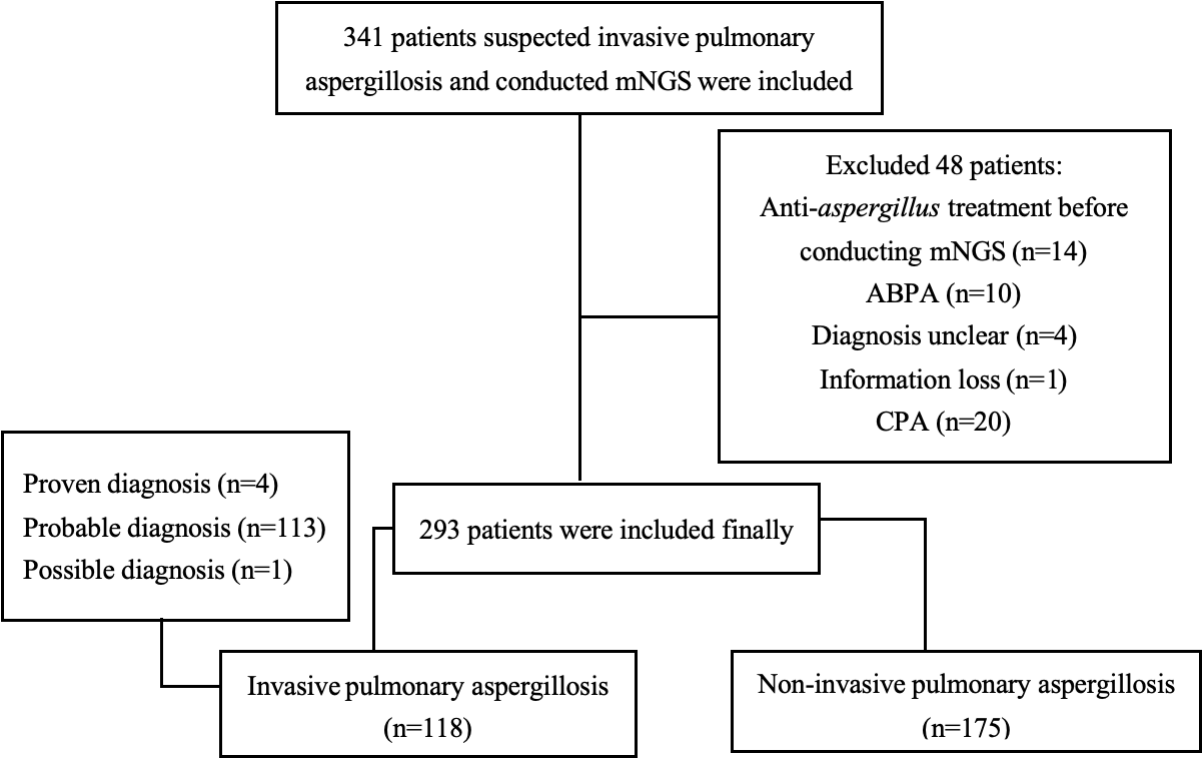
Study flowchart. mNGS, metagenomic next generation sequencing; ABPA, allergic bronchopulmonary aspergillosis; CPA, chronic pulmonary aspergillosis.

From October 2020 to February 2024, a total of 341 non-neutropenic patients with suspected IPA who underwent mNGS were enrolled. After excluding 48 ineligible patients, 293 were included in the final analysis, comprising 118 patients in the IPA group and 175 in the non-IPA group (**Figure 1**).

### 2.5 mNGS, GM test and culture

Samples for mNGS testing were sent to a qualified biotechnology company (Difei Medical Technology (Nanjing) Co., Ltd) which cooperated with each hospital included in our study. According to standard procedures, nucleic acid extraction and purification, library construction, high-throughput sequencing, bioinformatics analysis, and pathogen reports were performed. The details were as follows:

1. Nucleic Acid Extraction: Plasma was prepared from blood samples and circulating cell-free DNA (cfDNA) was isolated from plasma using the QIAamp Circulating Nucleic Acid Kit (Qiagen) according to the manufacturer’s protocols. Sputum, BALF and lung tissue were liquefied by 0.1% DTT (dithiothreitol) for 20 minutes at 56°C prior to extraction. DNA was extracted using the TIANamp Magnetic DNA Kit (Tiangen) according to the manufacturer’s protocols. The quantity and quality of DNA were assessed using the Qubit (Thermo Fisher Scientific) and NanoDrop (Thermo Fisher Scientific), respectively.
2. Library Preparation and Sequencing: DNA libraries were prepared using the Hieff NGS C130P2 OnePot II DNA Library Prep Kit for MGI (Yeasen Biotechnology) according to the manufacturer’s protocols. Agilent 2100 was used for quality control, and DNA libraries were subjected to 50 bp single-end sequenced on the DIFSEQ-200 platform.
3. Bioinformatics Analysis: An in-house developed bioinformatics pipeline was used for pathogen identification. Briefly, high-quality sequencing data were generated by removing low-quality reads, adapter contamination, duplicated and short (length<36 bp) reads. Human host sequences were identified by mapping to the human reference genome (hs37d5) using bowtie2 software (version 2.2.6). Reads that could not be mapped to the human genome were retained and aligned with microorganism genome database for pathogens identification. The microorganism genome database contained genomic sequences of bacteria, fungi, viruses and parasites.

The GM test was conducted by the clinical laboratories in each hospital using the double-sandwich enzyme-linked immunosorbent assay (Bio-Rad, RNOVBIO or Dynamiker Bio). Culture was also routinely performed by laboratories in each hospital, and the sputum or BALF samples were cultured on CHROMagar and incubated at 35°C for two (sputum) or three (BALF) days, and would be transferred to Sabouraud dextrose agar for further identification if *Aspergillus* was found. All centers followed the standard operating procedure and were supervised by the leading unit.

### 2.6 Statistical analysis

All statistical analyses were performed by IBM SPSS Statistics 25.0 (IBM, Armonk, NY). Quantitative variables are summarized by mean ± SD in normal distribution or median with interquartile range (IQR) in non-normal distribution, and qualitative variables are shown as numbers (percentages). The chi-square and the correction chi-square tests were used for categorical variables. Quantitative variables with normal distributions were compared with Student’s t-test, while non-normally distributed variables were compared with the Mann–Whitney U-test. An effect was considered to be statistically significant when the p-value was <0.05, and all significance tests were two-tailed.

### 2.7 Ethics

The study was approved by the institutional review board of the Nanjing Jinling Hospital (2020NZKY-012-01). Written informed consent was all obtained from patients who participated in this study.

## 3 Results

### 3.1 Baseline characteristics

Baseline characteristics of 118 patients with IPA (4 proven, 113 probable, and 1 possible cases) and 175 non-IPA patients are summarized in **Table 1**. The most common underlying disease in the IPA group was diabetes (23.1%), followed by bronchiectasis (22.0%), extrapulmonary malignancy (22.0%), hepatic or renal dysfunction (22.0%), circulatory system disease (19.5%), COPD (18.6%), lung cancer (15.4%), and tuberculosis (12.7%). Compared with the non-IPA group, COPD, lung cancer, and extrapulmonary malignancy were more prevalent in the IPA group (p<0.05). Among patients with suspected IPA, nearly one-quarter had a history of glucocorticoid use for more than three weeks. Although no statistical difference was observed, the proportion of glucocorticoid use was higher in the IPA group than in the non-IPA group (27.1% vs. 18.3%, p=0.073). Furthermore, the rate of admission to the intensive care unit was significantly higher in IPA patients than in non-IPA patients (41.5% vs. 15.4%, p<0.001).

**Table 1:** Baseline characteristics and underlying disease of IPA and non-IPA patients.

Characteristics	IPA (n=118)	non-IPA (n=175)	P value
Sex, male, n (%)	90 (76.3)	119 (68)	0.125
Age, years	68 (56~72)	59 (51~70)	0.001
BMI, kg/m2	21.08 ± 3.7	22.27 ± 3.9	0.007
Underlying pulmonary disease, n (%)	72	94	
bronchiectasis	26 (22.0)	46 (26.3)	0.407
COPD	22 (18.6)	13 (7.4)	0.004
lung cancer	18 (15.4)	14 (8.0)	0.048
previous or existing pulmonary tuberculosis	15 (12.7)	27 (15.4)	0.515
Underlying systemic disease, n (%)			
extra-pulmonary malignancy	26 (22.0)	16 (9.1)	0.002
diabetes	27 (23.1)	40 (22.9)	0.965
hepatic or renal dysfunction	26 (22.0)	31 (17.7)	0.360
circulation system disease	23 (19.5)	24 (13.7)	0.186
^#^Long-term use of glucocorticoid, n (%)	32 (27.1)	32 (18.3)	0.073
oral or intravenous	24 (20.3)	29 (16.6)	0.411
inhalation	8 (6.8)	3 (1.7)	0.054^*^
^#^Long-term use of immunosuppressant, n (%)	14 (11.9)	24 (13.7)	0.644
ICU patients, n (%)	49 (41.5)	27 (15.4)	<0.001

^*^Corrected chi-square; ^#^use of glucocorticoid (prednisone)≥20mg/d or immunosuppressant over 3 weeks; BMI, body mass index; COPD, chronic obstructive pulmonary disease; ICU, intensive care unit; IPA, invasive pulmonary aspergillosis.

### 3.2 Comparison of diagnostic performance of different microbiological methods for IPA

The diagnostic performance of various microbiological assays is presented in **Tables 2**, **3**. Overall, sputum culture and BALF culture showed comparable sensitivity and specificity (30.0% vs. 27.0%, 99.3% vs. 98.5%, p>0.05). Similarly, BALF GM assay demonstrated specificity comparable to serum GM, but its sensitivity was significantly higher than that of serum GM at both cutoff values (GM≥0.8 or 0.7 cutoff value, 64.4% vs. 24.1%; GM≥1.0 cutoff value, 55.8% vs. 16.7%, p<0.05).

**Table 2:** The positive rate of different microbiological testing methods in IPA patients.

Methods	Positive results/total patients	Positive rate	P value
Culture			
sputum	30/100	30.0%	/
BALF	24/89	27.0%	/
GM detection			
serum (≥0.7)	26/108	24.1%	/
serum (≥1.0)	18/108	16.7%	/
BALF (≥0.8)	67/104	64.4%	/
BALF (≥1.0)	58/104	55.8%	/
mNGS			
BALF	86/105	81.9%	0.004/<0.001 (mNGS vs. GM≥0.8/1.0)
sputum	6/9	66.7%	0.025^*^ (mNGS vs. culture)
blood	10/17	58.8%	0.008/<0.001^*^ (mNGS vs. GM≥0.7/1.0)

^*^Corrected chi-square; GM, Galactomannan; BALF, bronchoalveolar lavage fluid; mNGS, metagenomic next generation sequencing.

**Table 3:** Comparison of diagnostic efficiency of IPA among different microbiological methods.

Method	Sensitivity (%)	Specificity (%)	PPV (%)	NPV (%)	Positive-likelihood ratio	Negative-likelihood ratio	Youden index
Sputum culture	30.0	99.3	96.8	66.4	41.667	0.705	0.293
BALF culture	27.0	98.5	92.3	66.7	17.743	0.742	0.255
Serum GM (≥0.7)	24.1	95.5	78.8	64.4	5.325	0.795	0.196
Serum GM (≥1.0)	16.7	97.4	81.8	62.7	6.461	0.855	0.141
BALF GM (≥0.8)	64.4	92.7	85.9	79.1	8.849	0.384	0.571
BALF GM (≥1.0)	55.8	94.7	87.9	75.7	10.523	0.467	0.505
BALF mNGS	81.9	92.2	86.9	89.1	10.527	0.196	0.741
BALF mNGS/GM (≥0.8)	90.7	85.9	80.7	93.4	6.439	0.108	0.766
BALF mNGS/GM (≥1.0)	88.7	87.9	82.7	92.3	7.339	0.129	0.766
Low *aspergillus* reads (1-3)			83.3 (10/12)				
Low *aspergillus* reads of BALF (1-3)			77.8 (7/9)				

GM, Galactomannan; BALF, bronchoalveolar lavage fluid; mNGS, metagenomic next generation sequencing; PPV, positive predictive value; NPV, negative predictive value.

BALF mNGS was performed in 105 of the 118 IPA patients, with a positivity rate for *Aspergillus* detection of 81.9% (86/105). This rate was significantly higher than that of BALF culture (81.9% vs. 27.0%, p<0.001), BALF GM (81.9% vs. 64.4% at GM≥0.8, p=0.004; 81.9% vs. 55.8% at GM≥1.0, p<0.001), and sputum culture (81.9% vs. 30.0%, p<0.001). However, the specificity of BALF mNGS (92.2%) was comparable to that of these assays (92.7% or 94.7% for BALF GM, 98.5% for BALF culture, and 99.3% for sputum culture). Sputum mNGS was performed in only 9 IPA patients, with a positivity rate of 66.7% (6/9), which was significantly higher than that of sputum culture (66.7% vs. 30.0%, p=0.025). For blood samples, mNGS in IPA patients yielded a positivity rate of 58.8% (10/17), which was significantly higher than that of serum GM (58.8% vs. 24.1% at GM≥0.7, p=0.008; 58.8% vs. 16.7% at GM≥1.0, p<0.001). From a longitudinal perspective, the sensitivity of BALF mNGS was higher than that of blood mNGS (81.9% vs. 66.7%, p=0.006) and sputum mNGS (81.9% vs. 58.8%, p=0.502), but there was no statistical difference. In addition, BALF mNGS also had nice positive predictive value (PPV, 86.9%) and negative predictive value (NPV, 89.1%).

### 3.3 Value of combined diagnostic approaches

For the early diagnosis of IPA, we combined BALF GM and mNGS, which improved sensitivity to 90.7% (using BALF GM≥0.8 cutoff) and 88.7% (using BALF GM≥1.0 cutoff). This sensitivity was significantly higher than that of BALF GM alone (p<0.001) but did not differ statistically from BALF mNGS alone (90.7% vs. 81.9% for GM≥0.8, p=0.070; 88.7% vs. 81.9% for GM≥1.0, p=0.177). Additionally, the NPV of the combined BALF GM and mNGS approach was significantly enhanced, indicating its potential to reduce missed diagnoses.

Overall, BALF mNGS alone and the combination of BALF GM and mNGS showed superior diagnostic performance, with Youden indices of 0.741 and 0.766, respectively. A notable concern for clinicians is the interpretation of low *Aspergillus* read counts in mNGS results. In our cohort, the positive predictive value (PPV) for low *Aspergillus* reads across all specimens was 83.3% (10/12), while the PPV specifically for low *Aspergillus* reads in BALF mNGS was 77.8% (7/9).

### 3.4 Distribution of pathogens in IPA patients

**Figure 2:**
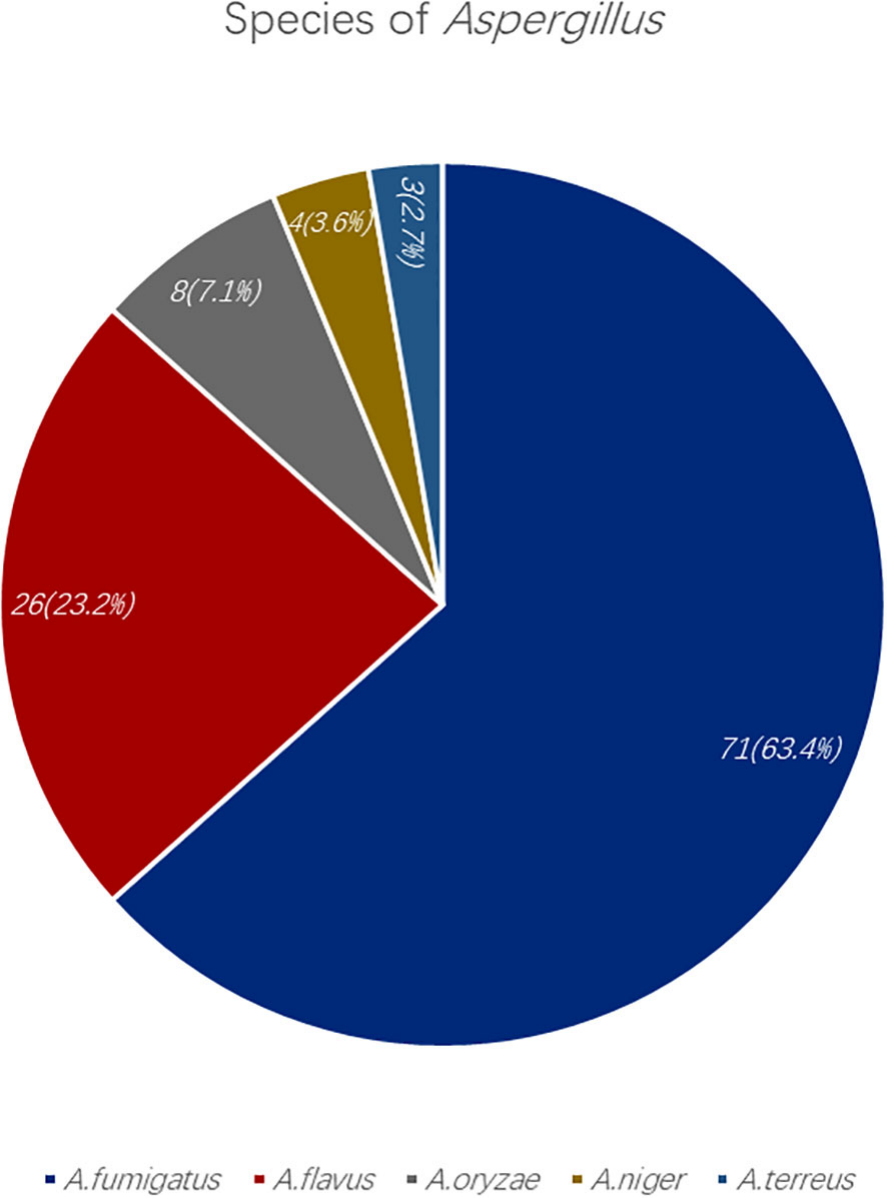
The distribution of different species of *Aspergillus* in the IPA group from our study.

**Figure 3:**
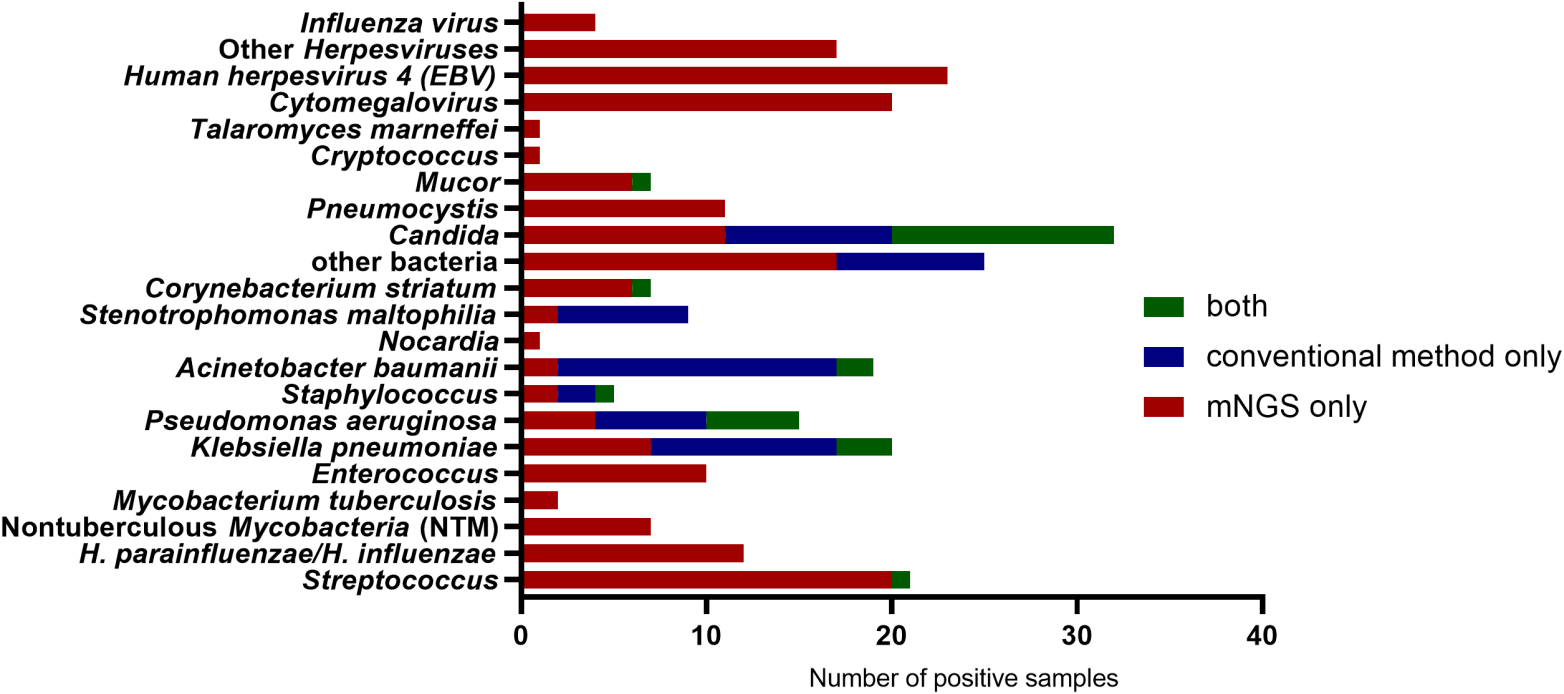
The distribution of suspected co-infection pathogens in patients with IPA. IPA, Invasive pulmonary aspergillosis.

As depicted in **Figure 2**, *A. fumigatus* was the most prevalent species among IPA patients (63.4%), followed by *A. flavus* (23.2%), *A. oryzae* (7.1%), *A. niger* (3.6%) and *A. terreus* (2.7%). Notably, *A. oryzae* frequently co-occurred with *A. flavus* in this cohort. Co-infections with other bacteria, viruses, or fungi were observed in 74.6% of IPA patients. *Streptococcus* and *Hemophilus influenzae* were common bacteria, and as for fungus, *Candida* was often detected and considered as colonization (**Figure 3**). Compared with conventional microbiological methods, mNGS enabled rapid and comprehensive identification of additional causative pathogens—particularly viruses, fungi, and fastidious or uncultivable organisms such as *pneumocystis* (n=11), *mucor* (n=6), *cryptococcus* (n=1), and *nocardia* (n=1).

### 3.5 Comparison of diagnostic timelines between patients with and without mNGS

**Figure 4:**
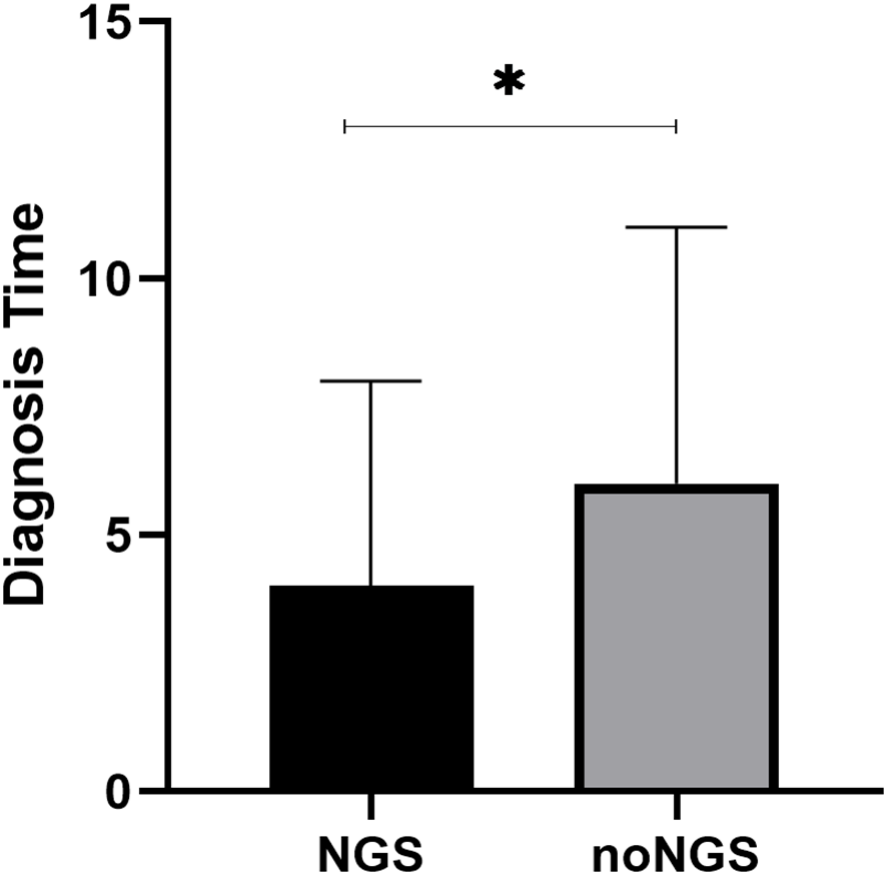
Compare of diagnostic time between patients with and without mNGS (4 days vs. 6 days, p=0.036). mNGS, metagenomic next generation sequencing.

Using data from the ChiCTR2200063453 registry, we identified 54 IPA patients who did not undergo mNGS, and compared their diagnostic timelines with those of IPA patients who underwent mNGS. The duration from hospital admission to IPA diagnosis was significantly shorter in the mNGS group than in the non-mNGS group (4 days vs. 6 days, p=0.036) (**Figure 4**).

## 4 Discussion

IPA is a severe fungal infection requiring early diagnosis to optimize outcomes. Notably, an increasing number of IPA cases are being reported in non-neutropenic patients lacking typical risk factors or distinct clinical manifestations, leading to frequent misdiagnosis or delayed identification. mNGS, a powerful emerging tool for infectious disease diagnostics, is increasingly integrated into clinical practice for IPA detection. This multicenter prospective study, enrolling 341 patients with suspected IPA, aimed to evaluate the clinical utility of mNGS in diagnosing IPA in non-neutropenic populations.

A retrospective analysis of pulmonary fungal epidemiology in China, synthesizing data from 16 studies, previously identified solid tumors, COPD, tuberculosis, and diabetes as the most common underlying conditions in affected patients—rather than hematological malignancies (You-Ning et al., 2011). Consistent with this, our study found that IPA patients predominantly had underlying diseases including diabetes, extrapulmonary malignancy, hepatic or renal dysfunction, circulatory system disease, COPD, lung cancer, and tuberculosis, aligning broadly with prior reports on non-neutropenic IPA cohorts (Liu et al., 2021; Tudesq et al., 2019). Recent evidence highlights IPA as a complication in severe influenza and COVID-19 patients (Latgé and Chamilos, 2019; Bassetti et al., 2021); however, due to constraints related to specimen collection and epidemic control measures, our cohort included only a small number of such cases. Long-term glucocorticoid use is well established as a risk factor for IPA (Donnelly et al., 2020; Agustí et al., 2003; Patel et al., 2011; Yao et al., 2024), and our data corroborate this: over one-quarter of IPA patients had a history of systemic glucocorticoid exposure, a proportion higher than in non-IPA patients. In intensive care unit (ICU) settings, risk factors for IPA include short- or long-course steroid use, broad-spectrum antibiotic therapy, COPD, acute respiratory distress syndrome (ARDS), liver failure, and the severity of organ dysfunction (Tudesq et al., 2019). In our study, nearly half of IPA patients required ICU admission—significantly more than non-IPA patients—emphasizing the need for heightened vigilance regarding *Aspergillus* infection in critically ill populations.

Microbiological evidence of *Aspergillus* infection is essential for the diagnosis of IPA. Culture and GM test have been demonstrated to be valuable for the diagnosis of IPA, although the specificity is considerable, the low positive rate is also a clinical problem. Multiple studies indicate that the sensitivity of sputum and BALF culture ranged from 29.0% to 43.3% and 9.6% to 36.4%, respectively (Liu et al., 2021; Lu et al., 2023; Zhou et al., 2017), which is in accordance with our study (30.0% and 27.0%), and the sensitivity of them was significantly lower than corresponding sputum mNGS (66.7%) and BALF mNGS (81.9%). Zhou et al. (2017) demonstrated that BALF GM sensitivity exceeds that of serum GM across all cutoff values, with comparable specificity; at cutoffs of 0.7 and 1.0, BALF GM sensitivities were 72.97% and 64.86%, respectively. In our study, using BALF GM cutoffs of 0.8 and 1.0 (in line with the latest guidelines (Donnelly et al., 2020)), sensitivities were slightly lower. This discrepancy may stem from differences in study populations. In our study, diabetes patients took a large proportion, and Lu et al. (2023) found that patients with IPA and diabetes had a significantly lower positive rate than patients with IPA but without diabetes, which may explain the low sensitivity of BALF GM in this study, but the mechanism needs to be further studied (Taghizadeh-Armaki et al., 2017).

Prior studies have reported that BALF mNGS exhibits a sensitivity of 66–92%, outperforming conventional microbiological assays, with greater diagnostic utility in immunocompromised versus non-immunocompromised populations (Zhan et al., 2023; Shi et al., 2023; Zhu et al., 2023; Liu et al., 2024). In our multicenter prospective cohort, the sensitivity of BALF mNGS was 81.9%, which was significantly higher than that of BALF GM, while the specificity was similar. Subgroup analyses revealed sensitivities of 85.2% and 81.8% in diabetic and COPD patients, respectively. Combining BALF mNGS with BALF GM (using cutoffs of ≥0.8 or ≥1.0) increased sensitivity to 90.7% or 88.7%, respectively, which may facilitate earlier IPA diagnosis, albeit with a modest reduction in specificity. Based on the Youden index, both BALF mNGS alone (0.741) and the combined approach (0.766) showed strong diagnostic value. Additionally, BALF mNGS and GM assays exhibited high negative predictive values, which may help reduce missed diagnoses. A common clinical challenge is interpreting mNGS reports with low *Aspergillus* read counts (1–3 reads), which often leads to diagnostic uncertainty. In our study, among 12 IPA patients with low *Aspergillus* reads in mNGS results, 10 were confirmed as true positives, yielding a PPV of 83.3%.Specifically for BALF mNGS, the PPV for low *Aspergillus* reads was 77.8%. These findings suggest that in patients with suspected IPA, even low *Aspergillus* read counts in mNGS results may strongly support an IPA diagnosis.

Overall, it seems that BALF shows better diagnostic performance than sputum and blood in the detection of *Aspergillus*, whether by culture or GM testing. This aligns with a study of IPA in hematological patients, where BALF-based GM, PCR, and mNGS all demonstrated higher sensitivity than blood-based tests (Xu et al., 2025), supporting BALF as the preferred specimen for IPA diagnosis when available. However, blood is always the most common specimen with the advantage of causing little damage in clinical, especially for patients who cannot undergo bronchoscopy. Serum GM has the feature of high specificity but low sensitivity, especially for nonneutropenic patients with limited angioinvasion. mNGS can increase the positive rate by detecting microbial gene fragments in plasma. A study showed that in blood samples, mNGS had the highest sensitivity (71.9%) for IPA diagnosis in neutropenic patients (Xu et al., 2025). Another two-center cohort study about COVID-19-associated pulmonary aspergillosis (CAPA) indicates that the sensitivity of plasma mNGS could be up to 83% with a specificity of 97% for the diagnosis of CAPA. In our study, involving a wide population, the sensitivity of blood mNGS was 58.8%, lower than the above studies, but still significantly higher than that of serum GM. The higher positive rate of serum mNGS for CAPA may be related to the vascular invasion during the late stage of COVID-19 infection (Feys et al., 2024). It is a pity that we did not include this population and needs further study.

*A. fumigatus* is the most common species of *Aspergillus* causing IPA (Latgé and Chamilos, 2019), which is in accordance with our study. The following common species were *A. flavus*, *A.oryzae*, *A.niger* and *A.terreus*, which is similar to some previous studies (Zhan et al., 2023). Besides, we found that *Aspergillus oryzae* was often co-infected with *Aspergillus flavus*, the reason might be their phylogenetic, genomic, and metabolic homogeneity according to a basic study and they even thought that they may indeed belong to the same species (Han et al., 2024).

An important added advantage of mNGS is its potential to detect rare fungi that may be missed or misclassified by traditional microbiological test such as *Mucor* and *Cryptococcus* (Hoenigl et al., 2023; Hogan et al., 2021). Particularly for pulmonary mucormycosis, whether host factor, clinical manifestation or imaging, it is exactly similar with IPA, mNGS is beneficial for detecting *Mucor* early and enabling timely treatment. In our cohort, 6 patients had concurrent infections with *Aspergillus* and *Mucor*, 5 of these cases were identified solely by mNGS. Overall, the pathogen detection rate of mNGS was significantly higher than that of conventional culture methods.

Early initiation of treatment is critical for improving the prognosis of IPA. Most mNGS platforms could complete analysis from sampling to the final results during 24-72h (Han et al., 2019). We analyzed the diagnostic time between IPA patients undergoing mNGS and those without, and found that the diagnostic period of patients undergoing mNGS was significantly shorter than that of patients without, which was similar to some studies (Liu et al., 2024). Nevertheless, several limitations of mNGS persist in clinical application. First, specimen processing profoundly impacts result accuracy. For pathogens such as fungi with thick cell walls or intracellular bacteria, insufficient cell lysis can compromise DNA extraction efficiency, leading to false-negative results. Conversely, excessive disruption may degrade specific pathogen gene fragments, hindering their detection. Thus, optimized sample processing protocols are imperative. Second, mNGS detects a broad spectrum of microorganisms, including colonizers and contaminants, complicating the differentiation between true pathogens and non-pathogenic entities. Additionally, the high cost of mNGS remains a substantial barrier, particularly for patients in resource-limited settings.

There were some limitations in our study. First, the number of samples for blood mNGS and sputum mNGS was small, which may result in potential selection bias. In future comprehensive studies, we will expand the study population to address this limitation. Second, there was a lack of COVID-19 patients with IPA due to the limitations of specimen collection and epidemic control, leading to an incomplete population. Future studies will aim to address these gaps through more comprehensive design and implementation.

## 5 Conclusion

In conclusion, mNGS, as an emerging technology, demonstrates substantial value in the identification of IPA. Its sensitivity was significantly higher than current conventional microbiological tests, while maintaining comparable specificity. Among various specimen types, BALF seemed to be a superior choice, exhibiting robust sensitivity and specificity relative to sputum and blood samples. For patients unable to undergo bronchoscopy, however, mNGS performed on sputum or blood remains a more effective alternative to conventional methods. Notably, the combination of BALF mNGS with GM testing yields excellent sensitivity and negative predictive value. Additionally, mNGS offers distinct advantages in detecting co-infections, facilitating timely treatment adjustments, and shortening the diagnostic timelineics,s,ldt that further underscore its clinical utility in managing IPA.

## Data Availability

The raw data supporting the conclusions of this article will be made available by the authors, without undue reservation.
